# Exploring Social Media Group Use Among Breastfeeding Mothers: Qualitative Analysis

**DOI:** 10.2196/11344

**Published:** 2018-11-05

**Authors:** Kara Renee Skelton, Retta Evans, Jenna LaChenaye, Jonathan Amsbary, Martha Wingate, Laura Talbott

**Affiliations:** 1 Department of Health, Behavior and Society Johns Hopkins University Baltimore, MD United States; 2 University of Alabama at Birmingham Birmingham, AL United States

**Keywords:** social media, milk, human, breastfeeding

## Abstract

**Background:**

Breastfeeding is well known as the optimal source of nutrition for the first year of life. However, suboptimal exclusively breastfeeding rates in the United States are still prevalent. Given the extent of social media use and the accessibility of this type of peer-to-peer support, the role of social networking sites in enabling and supporting breastfeeding mothers needs to be further explored.

**Objective:**

This study aimed to leverage mothers’ attitudes and behaviors of social media usage to understand effects on breastfeeding outcomes.

**Methods:**

Participants were recruited from 1 probreastfeeding social media group with over 6300 members throughout the United States. Online focus group discussions were conducted with 21 women; interviews were conducted with 12 mothers. Qualitative data were aggregated for thematic analysis.

**Results:**

Participants indicated that the social media group formed a community of support for breastfeeding, with normalizing breastfeeding, empowerment for breastfeeding, resource for breastfeeding, and shared experiences in breastfeeding as additional themes.

**Conclusions:**

According to participants, social media groups can positively influence breastfeeding-related attitudes, knowledge, and behaviors as well as lead to longer duration of breastfeeding. The results of this study should be taken into account when designing interventions for breastfeeding mothers.

## Introduction

### Background

Major health organizations, including the American Academy of Pediatrics, World Health Organization, and Academy for Breastfeeding Medicine, recommend, at minimum, exclusively breastfeeding for the first 6 months of life, with continued breastfeeding for at least 1 year and thereafter as mutually desired by both mother and infant [1-3]. Although breastmilk has been well known as the optimal source of nutrition for infants for decades, research continues to accumulate on the benefits of breastfeeding for the mother-infant dyad, building an extensive scientific database of high-level research consisting of qualitative and quantitative studies.

According to the Centers for Disease Control and Preventions’ 2018 Breastfeeding Report Card, approximately 4 out of 5 (4/5, 83%) infants born in 2015 were ever breastfed, with rates increasing from previous years. In addition, an estimated 57.6% of infants were breastfed at 6 months. However, less than half of those infants (24.9%) were exclusively breastfed at 6 months [4]. Breastfeeding disparities exist for southeastern states, where breastfeeding initiation rates are as low as 63.2%, and 6-month breastfeeding exclusivity at a mere 13.0% for Mississippi. For southeastern states, the highest prevalence of breastfeeding at 6 months occurred in Georgia (55.5%), which is lower than the US national average [4]. Furthermore, Healthy People 2020 has specific Maternal, Infant, and Child Health objectives (MICH) for breastfeeding, with MICH-21.5’s focus on breastfeeding exclusively through 6 months [5].

A recent, extensive meta-review of 28 systematic reviews and meta-analyses reasserted the most known and agreed upon effects of exclusively breastfeeding for the infant: lower infectious morbidity and mortality, higher intelligence than those who are formula fed, and protection against later-in-life development of overweight and diabetes, to name a few [1,6,7]. In addition to the direct benefits of exclusively breastfeeding for the infant, there are also benefits gained by the mother from exclusively breastfeeding, which include protection against breast cancer, delayed onset of ovulation, and reduction in both ovarian and breast cancer risk [6]. A recent cost-benefit analysis for breastfeeding estimated that the *scaling up* of breastfeeding could prevent 823,000 child deaths and 20,000 breast cancer deaths per year worldwide [6]. These benefits associated with breastfeeding are key components for health promotion and disease prevention for the mother-infant dyad [8-11], which is why it is important for women to be able to not only attempt breastfeeding but also be successful at it.

Access to social support during the perinatal and postpartum periods have been linked to better maternal health and child development outcomes as well as increased relationship satisfaction (for partner-to-partner as well as parent-child interactions) [12,13]. Conversely, lack of social support is often cited as a reason for breastfeeding cessation [14-17]. Peer support can come from family, friends, or even other mothers who are strangers [17,18], and this support can occur in-person or across virtual modalities [17,19,20]. A recent meta-analysis of social support interventions for breastfeeding mothers has shown to increase breastfeeding initiation by 86% and exclusive breastfeeding by 20% [21]. The US Preventive Services Task Force recommends peer support as 1 of the 3 recommended types (professional support and formal education being the other 2) during pregnancy and after birth to support breastfeeding [22].

In recent years, research around the juncture of motherhood and technology has grown significantly [13,23]. Social media outlets have been used to spread health-related messages as well as to provide a *forum* for those seeking health information. The main advantage of using social networking sites (SNSs) concerning health information is that they enable the widespread interaction of users as both receivers and providers of health information and knowledge [24]. Although the number of studies around social media use, social capital, and technology has increased, there is still much to be explored in this emerging realm—especially regarding MICH.

### Objectives

The exponential growth of user-generated content embedded within SNS elicits a need for a further understanding of communication dynamics involved in these online forums [25]. Although numerous studies have provided a foundation for evidence of SNSs as community building and even stated the use of groups on Facebook as a pathway for community interaction [23], there is a lack of knowledge in the scientific community about how breastfeeding mothers influence other mothers online and how these influences impact mother’s and infant’s health outcomes. In addition, there exists (minimal, at best) research on how communication between breastfeeding mothers who use SNS supports breastfeeding. As it is well known that social relationships and support play a critical role in breastfeeding-related behaviors, social media usage is a topic of importance [26,27].

Furthermore, the use of virtual communities within SNSs for knowledge sharing is only recently being studied [28]. Individual’s personal values can often serve as motivation for knowledge sharing in the absence of personal familiarity or assumptions of direct reciprocity, indicating social capital plays a significant role in knowledge contribution within the online realm [29]. Despite the growth in literature regarding knowledge sharing within SNS, current research on breastfeeding-related behaviors and outcomes within the realm of SNS is narrow, focusing on extrinsic motivators, already known benefits of breastfeeding, or familial or partner support. There exists immense opportunity for recently emerged technologies, such as social media groups on SNSs, to provide interaction, support, and information to breastfeeding mothers. However, to date, there exists very little information regarding breastfeeding mothers’ use of social media groups and its impact on knowledge, attitudes, and behaviors. To address this gap, the proposed study aims to leverage mothers’ attitudes and behaviors of social media usage to understand effects on breastfeeding outcomes.

The qualitative findings shared here are part of a mixed-methods study to comprehensively explore mothers’ behaviors and attitudes of social media group usage toward the online platform as a means of increasing breastfeeding uptake. The goal of the qualitative phase of the study is to gain meaningful, in-depth insight into the mindset of mothers. The following research question guided the qualitative strand of the study: How does social media group usage support breastfeeding mothers?

## Methods

### Study Design and Setting

The results of the qualitative strand of this study were used to guide the development of a quantitative tool to assess breastfeeding mothers’ social media group use and breastfeeding-related attitudes, knowledge, and outcomes. Participants were selected purposefully through a snowball sampling design. Women who were members of a Facebook probreastfeeding social media group were recruited via a post on the group wall in the fall of 2017. This group was selected because of the large number of members (>6300), their *probreastfeeding* approach (as designated by the title of the group), and accessibility to the group (US-based). This Facebook group originally stemmed from an in-person support group based at a mid-sized hospital in Birmingham, Alabama. However, there are no restrictions for joining the group: *any and all breastfeeding moms are welcome*, according to the Facebook group description. There are 5 *administrators* of the group, some of whom have International Board Certified Lactation Consultant (IBCLC) certification and others who do not have any professional training but are experienced in breastfeeding, either from feeding their children or from other experience (eg, work experience as a labor and delivery or neonatal intensive care unit nurse or from being a lactation counselor or dietician). Study participation was limited to women who were pregnant and intended to breastfeed, were currently breastfeeding, or who had recently weaned their infant in the past 3 years.

### Study Participants

After screening for inclusion criteria, eligible participants were asked to participate in 1 of the 3 online focus group discussions (FGDs) or one-on-one interviews. Recruitment was conducted via wall posts within the group that asked mothers to participate in either a focus group or interview. Mothers who responded to recruitment posts were first asked to participate in focus groups. After focus groups were filled, respondents were then asked to participate in one-on-one interviews. All slots (as determined by when saturation would be achieved) were filled for both focus groups and interviews within 48 hours, so the post was then deleted. Of the 37 women recruited for online FGDs or interviews, all were eligible to participate. However, only 29 participants provided consent. A total of 21 participants participated in the online FGDs and 12 mothers participated in interviews; 4 mothers participated in both the online FGDs and interviews for validity purposes.

### Data Collection

After informed consent was obtained, online FGD participants were randomized to either the first, second, or third online FGD. Participants were then added to a secret online group and asked to complete a demographic questionnaire before participating in the online FGDs. The online FGDs were asynchronous, and participants were given 4 days to read and respond to the initial post as well as to respond to and interact with others in the group. Detailed methodology, including reflection on the utilization of this methodology with mothers, is published elsewhere [30]. The intent of the interviews was to generate greater depth on themes brought up in the online FGDs. As such, the online FGD analysis guided the development of the interview instrument. The interview instrument was designed to be open-ended and to elicit thoughts, feelings, and experiences about social media use and breastfeeding (eg, How do you think the probreastfeeding group has impacted your breastfeeding relationship? What about the other social media groups?, Discuss a time that a social media breastfeeding group has impacted a decision or choice you made in regards to breastfeeding, What are some barriers or pitfalls to using social media to post or interact with other mothers about breastfeeding?, and How would you describe the information posted in probreastfeeding group in regards to accuracy?). This guide was developed to be comprehensive of themes derived from the online FGDs, but open-ended enough to allow interviewees to describe their experiences. All interviews were performed after informed consent was obtained and demographic questionnaires were completed. Participants were compensated with a US $10 Amazon gift card for their participation. Both online FGDs and interviews were conducted with the University of Alabama at Birmingham Institutional Review Board approval and oversight (REC300000306).

### Data Analysis

Online FGDs and interview data were aggregated for analysis. The combined data transcript was then analyzed using Nvivo 10 (QSR International) for in-depth thematic analysis [31]. KRS coded the data and if she was unclear on a code, this was decided by the second coder. During initial coding, in-vivo coding was used for each phrase of the transcript, which was conducted by the researcher. The main reason for selecting an in vivo approach to coding was to stay *true* to the data, as this approach summarizes key phrases using participants’ own words [32]. This approach is also advocated for within the framework approach for qualitative research analysis [32]. KRS also reviewed the coded data according to each theme and created a preliminary analysis results document to be shared with participants. This member checking was conducted to verify if the researchers’ interpretation of the data was accurate. Themes were accepted by all 7 participants who were invited for member checking. The datasets generated and analyzed during this study are not publicly available as the authors do not have a website to publicly display them, but are available from the corresponding author on reasonable request.

## Results

### Demographics

At the time of enrollment, 2 women were currently pregnant (1 had previously breastfed and 1 was a first-time mother), 25 women were currently nursing, and 4 had weaned their child in the past 12 months. Other sociodemographic characteristics of participants are shown in Table 1.

Overall, 86% (25/29) of mothers selected their primary feeding method as exclusively breastfeeding (a mixture of at-breast feeds and breast milk bottles with no formula supplementation), with 16% (4/29) of these mothers feeding via at-breast feeds only and only 8% (2/29) exclusively pumping (infant only receives breastmilk from bottles). In total, 4 mothers reported ever providing supplementing formula; no infants were receiving formula supplementation at the time of the study. When asked how long they had been in the probreastfeeding social media group, most (18/29, 62%) had been in the group for more than 12 months, with 13% (4/29) and 34.% (10/29) being in the group between 6 and 12 months and less than 6 months, respectively. Interaction with the group varied: 75% (22/29) participants said they give advice and ask questions regularly within the group and 17% (5/29) stated that they do not interact regularly. For those who do not interact regularly, participants reported they searched in the group before posting or had recently weaned their infant as reasons why they were not currently active in the group.

**Table 1:** Demographic characteristics of online focus group discussions and one-on-one interview participants (N=29).

Demographic characteristics		Statistics
Age in years, mean (range)		29.7 (23-40)
**Race, n (%)**		
	African American	3 (10)
	American Indian	1 (3)
	White	25 (86)
**Education, n (%)**		
	High school diploma or some college	11 (38)
	Bachelor’s degree (4 year)	10 (35)
	Master’s degree	6 (21)
	Professional degree (Juris Doctor and Doctor of Medicine)	1 (3)
**Working status, n (%)**		
	Full- or part-time	25 (86)
	Not working	4 (14)
**Type of birth, n (%)**		
	Vaginal or vaginal birth after Cesarean	22 (76)
	C-section	7 (24)
Infant ever admitted to neonatal intensive care unit, n (%)		0 (0)
**Interaction with social media group, n (%)**		
	Ask questions	22 (76)
	Give advice	22 (76)
	Does not interact regularly	5 (17)

Participants’ attitudes, behaviors, and experiences with social media groups while breastfeeding were analyzed. The analysis resulted in 1 overarching theme of community, with the following supporting themes: (1) normalizing breastfeeding; (2) empowerment for breastfeeding; (3) resource for breastfeeding; and (4) shared experiences in breastfeeding. These themes are elaborated in detail below, providing quotes from participants for further harmonization and understanding.

### Normalizing Breastfeeding

Mothers felt the group helped to combat stigma by normalizing breastfeeding, which emerged as a supporting theme. A large majority of posts were discussing how stigmatized breastfeeding still is, both on social media and in the real world. The stigma or fear of judgment extended beyond the realm of the public sphere and into the mothers’ families. Participants described the process as “isolating”:

Breastfeeding is still taboo in public. Sometimes our husbands or significant others or family members aren’t supportive.

The theme of unsupportive friends and family members reigned throughout both online FGDs and one-on-one interviews:

Attitudes from friends and family definitely impacted my breastfeeding relationship. I felt unwanted a lot when visiting my in-laws and almost stopped nursing several times because of it. I feel like their attitudes also made me act unfairly towards my daughter. I often wouldn’t let her control how long the session lasted because I knew they would come and ask if we were done yet.

Although a majority of mothers felt like their friends and family were not supportive of breastfeeding, a few reported that their friends and family were supportive:

I do however have one friend who has done nothing but encourage and support me. Her attitude helped me feel secure in my decisions until I decided to wean and she encouraged me to continue. She was still supportive when I went through with it though.

Again, most participants felt that breastfeeding was still stigmatized, especially certain aspects of breastfeeding or how a mother chose to breastfeed (eg exclusively breastfeeding, exclusively pumping, or supplementing while breastfeeding). One participant commented:

A breastfeeding barrier that seem to be the most common for me is unsupportive people. Whether that’s from comments on social media posts on breastfeeding, a family member, or a stranger giving me the side eye while nursing in public.

In addition to breastfeeding, mothers mentioned breastfeeding in public, exclusively pumping, continued breastfeeding (past 12 months), cosleeping, nursing at night, and supplementing while breastfeeding as all having their own *taboo*. As 1 participant stated:

Women are afraid of being shamed for breastfeeding. Whether it’s for not being covered properly or the age of the child. I for one have experienced negative comments from some regarding nursing my 15 month old. Because of this, I don’t readily advertise or discuss my breastfeeding relationship outside of the groups.

It was discussed that within the group, most mothers felt like the group itself tried to normalize breastfeeding for mothers. This included expectations—what to expect at the beginning of the breastfeeding relationship, during the weaning process, and everything in between. The group was described as providing participants the opportunity to “understand what to expect and what’s ‘normal’” and “feel that, as with anything else in life, if expectations are properly set, everyone will be more satisfied with the experience.” This information sharing generated positive outcomes within the mothers’ experiences and overall approach of the activity:

It affects my attitude because it helps me know what to expect and, if I know what to expect and what’s “normal,” I am better able to accept what’s going on. For example, knowing that cluster feeding is a normal thing, it didn’t stress me out and cause frustration.

Another mother stated:

Since there were so many other moms in the group, I felt like that [itself] made breastfeeding seem more normal, especially coming from a family where not one person has breastfed.

Mothers felt like this normalizing within the group contributed to the success of their breastfeeding outcomes.

### Empowerment for Breastfeeding

Almost all mothers also talked about the sense of empowerment they felt from the group. They discussed this in the form of confidence, empowerment, and support. For 1 mother, the group played a vital role in her breastfeeding relationship:

With my oldest son, I did not breastfeed at all so I knew nothing. However, I knew I wanted to breastfeed this time around, so I joined several breastfeeding groups to learn as much as I could. I felt very overwhelmed and scared, but after reading others’ experiences and asking questions I felt much more confident. I wasn’t sure how long we’d make it, but we are at 8 months and going strong!

Mothers also discussed the high level of accountability in the group for encouraging one another. Some even stated that their breastfeeding duration or breastfeeding goals changed because of being in such a supportive and empowering environment. This was a common theme throughout both online FGDs and one-on-one interviews:

At the very beginning of my breastfeeding journey I was experiencing an extreme amount of pain. I wanted so badly to quit. The support I received from a social media group was invaluable. I was encouraged to never quit on a bad day. I never quit, because of the encouragement I got [from the group].

Another topic brought up in many of the online FGDs and one-on-one interviews was nursing in public and the group’s role in encouraging mothers to overcome this barrier. Many mothers talked about how supportive the social media group was for nursing in public, empowering and encouraging women to not be ashamed to feed their babies in public spaces, including parks, shopping centers, restaurants, and others’ homes:

I am more willing to nurse in public than I would have been without being a part of these social media groups. I am very conservative but I now have nursed my son while shopping and speaking to a sales clerk- something I would never have dreamed of until I felt empowered by all of the ladies in these groups!

Enhanced confidence was also brought up as a result of being in the group, going along with the theme of empowerment. From first-time mothers to experienced breast feeders, the majority of participants stated the group helped them to alleviate concerns or self-doubt they had regarding breastfeeding. Issues around having enough milk supply, supplementation of formula, use of prescription medication, and going back to work were all brought up in regards to maternal confidence. However, mothers reported that the social media group helped them feel empowered and confident about these issues after interacting with other members:

Largely because of what I was reading in [the social media group] I gained the confidence to allow my son to nurse on demand, as opposed to trying so desperately to adhere to a nursing schedule. I also decided to wait until a week or so before I go back to work to begin preparing my pumped stash. Outside of these two decisions, I have gained confidence and affirmation about the decision to nurse in general and guidance on so many questions/concerns/doubts I’ve had as a first-time mom.

### Resource

Mothers perceived the social media group to serve as a resource for breastfeeding mothers, providing real-time and accurate information for all things breastfeeding. Mothers in the study reported that just knowing the group existed to ask questions helped to alleviate their stress. Some mothers did not know any breastfeeding mothers, so the group served as a *pool* of potential mentor mothers to ask:

It benefitted me by having a resource for which I could ask literally any question under the sun related to breastfeeding, and I would have an answer and an explanation within hours, sometimes even within minutes. The ability to post on the group with questions and the peace of mind it gave me just knowing that it was there was very meaningful for me during my journey.

This value of information was further enriched by the level of availability to mothers, unlimited by time or access restraints:

Posting on social media groups will benefit us by making solid researched information, as well as personal experience from other moms, readily accessible. With no mothers in my own family who breastfed, the number of women to whom I can ask questions is very limited. Social media broadens that pool.

Mothers reported that the group was helpful at all hours of the day and night, where people could receive *real-time* answers. The real-time answers mothers reported to be helpful for sustaining their breastfeeding relationship covered a variety of topics and issues, stating:

I was really concerned about how much pumped milk to leave my baby when I returned to work. People flooded me with knowledge and charts to help calm my fears and helping my return to work become easier.

The social media group was reported to be a place of support and comfort when mothers did not know what to do or where to turn. This is prevalent throughout the data, but mothers discussed how after interacting with the group (through seeking advice, reading previous posts, or just interaction) their breastfeeding outcomes were positively impacted:

Well, when my child was going through his first growth spurt, he ate every single hour through the night. The next morning my mother-in-law expressed concern that my milk production was low or not keeping him full and asked if I wanted to supplement formula. Without the groups I’m in, I probably would've supplemented but because I could post and ask what to do, I found out it was completely normal and now we are on week 11 of breastfeeding.

This experience was common among participants, with outcomes stretching from basic nutrition to practical matters:

The [social media] group influenced me to still nurse as much as possible when our pediatrician recommended supplementing, they helped me know when my baby was gaining enough weight, they helped me decide how many times to pump at work, how many and what size bottles to send to daycare, etc.! I have learned so much!

### Shared Experiences

A common theme brought up was 1 aspect of the group mothers really appreciated—shared experiences. Mothers discussed they felt more trusting and able to understand the advice given within the group, as it came from other breastfeeding mothers. These mothers had gone through the same struggles and triumphs as others and were able to impart their knowledge onto others who were experiencing a similar situation:

These groups help me make decisions based on hearing experiences from a large group of women. As mothers, we are constantly questing ourselves because we do not want to mess up our children. These groups help me learn from others so that I can avoid some practices that might not be as effective as others.

Also reported in the online FGDs and one-on-one interviews was that mothers knew the value of having access to people with shared experiences. Most reported they did not know other mothers who were breastfeeding or had previously breastfed, so having the ability to ask advice and seek help from those who had gone through the experience in the group was a critical component of their successful breastfeeding relationship. Mothers discussed that they felt the shared experiences of others both comforting and empowering at the same time, stressing that “Nothing replaces training like experience. And doctors, and nurses, receive so little training in breastfeeding. Having the group as a resource is amazing” as well as simply “realizing I am not alone, in both the struggles and successes.”

Others felt the social media groups helped them to feel more empathy and compassion for other mothers. Through shared experiences and shared struggles during the breastfeeding journey, mothers felt they were able to connect with other mothers:

I feel like I’m able to be more compassionate and have more empathy toward all moms, because, through stories on the group page, I learned about moms dealing with multiple bouts of mastitis, baby biting, thrush, blebs and blisters, low pump supply, etc. Because I never experienced those things first hand, I feel like I wouldn’t be as understanding toward others having difficulty because I didn’t know it could be so hard for some.

### Community

The 1 overarching theme discussed in the online FGDs was *community*. Participants felt like the probreastfeeding social media group was a place where they felt a bond with other mothers and where they were understood. Some participants noted the group brought strangers together around 1 topic and united them. Participants described their relationship with the group as being “always nice to have a place to go where you are ‘understood’” as well as appreciating the group’s ability to “normalize not only breastfeeding but also the troubles that surround breastfeeding moms. It brings us together!”

Participants also brought up a strong sense of confidentiality within the community and current members of the community. They felt a strong sense of trust and nonjudgment from a group composed predominantly of strangers. However, participants said they would be hesitant to seek advice or help from the group if they had people they knew within the group. Coworkers, family, and even close friends were mentioned by participants within this context. As 1 participant shared:

I would be much less inclined to seek help from the group if I had coworkers that were also group members. I could see myself being too embarrassed to ask for help from people who know me, simply as a matter of pride.

A large portion of the discussion for both online FGDs and individual interviews integrated the trust and confidentiality within the social media group, indicating that these types of groups may be a rich place for knowledge sharing.

## Discussion

### Principal Findings

The sense of community and shared experience as well as overall support in the breastfeeding practice were major themes that emerged. Furthermore, findings from this qualitative study elicit the notion that certain probreastfeeding social media groups could be considered a pillar of support for breastfeeding mothers, which is consistent with findings that mothers seek support for breastfeeding through a variety of channels [12,16,17,33]. These channels include in-person support groups, mobile apps, and online forums. Online support mechanisms, including mobile apps and social media, have only recently been explored for utilization during the postpartum periods [34,19,35], and even more recently for breastfeeding support [16,19,20]. As such, there is miniscule information on how social media groups support breastfeeding mothers; these results and findings shed light on topics not previously covered through traditional *mothering* groups and bring up novel areas for providing support to breastfeeding mothers.

Another main theme discussed in the online FGDs and interviews was normalizing breastfeeding. This included discussions on how the probreastfeeding social media group tried to iterate the normalcy of breastfeeding in public, exclusively pumping, continued breastfeeding, cobreast sleeping, and night nursing, to name a few. Participants brought up the fact that they often felt stigmatized within the real world in regard to breastfeeding, but did not feel any stigma within the social media group. Although literature has shown that the stigma associated with breastfeeding in public has been associated with lower breastfeeding rates, our results show that the mothers within this social media group empower one another to overcome their fears and tackle nursing in public *breast-on* [36]. Although most mothers reported not knowing what to expect during the breastfeeding journey, almost all participants said the group helped to define realistic expectations and what *normal* means for breastfeeding mothers. Along with helping to establish and maintain expectations, this social media group was found to help *normalize* breastfeeding, having a profoundly positive impact on the breastfeeding journey.

Organically brought up by mothers was the sense of empowerment for breastfeeding they received from their interaction with the social media probreastfeeding group. This empowerment came to fruition in the form of confidence, empowerment, and generalized support for breastfeeding. There was a very high level of support and trust within this social media group, which led mothers to discussing more personal and sensitive topics—disclosing they shared more personal information within the group than they shared with their pediatricians or obstetricians. Mothers reported having access to, being able to interact with, and ask questions to those who had already gone through the same struggles and triumphs during the breastfeeding relationship was an incredible asset within the probreastfeeding social media group. This led to mothers disclosing they trusted other mothers’ advice within the probreastfeeding social media group more than they trusted their pediatricians’ or obstetricians’ breastfeeding-related advice. Pediatricians are not known as *experts* for breastfeeding; there are others (eg, IBCLCs, lactation consultants, and registered dieticians) who have extensive training and certifications to assist breastfeeding mothers. With the existence of these experts becoming more well known, it is not surprising that mothers are not trusting their pediatricians for breastfeeding advice. This also touches on the theme of peer support, which has been shown to help breastfeeding outcomes [12,16,22,33]. From mothers’ discussions of their interactions, it became clear that access to other breastfeeding mothers was a key supporting factor for the mother-infant dyad. It was also reported that through this interaction, mothers became more empathic and compassionate toward other breastfeeding mothers, reporting they were able to connect more with others, both inside and outside the social media group. This shows the importance of dynamic relationships and peer support throughout the breastfeeding process, especially for the formation of trust, which can help mothers adopt breastfeeding recommendations.

Social media groups, in general, were found to be a resource for breastfeeding mothers. However, mothers felt the degree of accuracy of information varied among social media groups. When mothers discussed the *probreastfeeding* social media group, they elaborated on not only the reliability of the information but also on how much they loved having access to *real-time* information. For example, mothers were able to ask a question at 2 am and get an almost immediate response from another breastfeeding mother who was up. Mothers reported this *real-time* resource as being invaluable to them—rather than having to search multiple websites for a specific answer or wait until a pediatrician or obstetrician visit, mothers were able to get fast and valid information from a variety of people—including IBCLCs and mothers who had already experienced the issue. It is critical to discuss the rapport of the social media group when discussing social media groups as a resource for breastfeeding mothers, as this can lead to trust or distrust among the members. Findings from other studies show that online support can be helpful for not only parenting [27,37] but specifically for breastfeeding [19,37].

In a broader context, it is important to bring up the distinct differences brought up between *mom groups* or *mothering groups* and *probreastfeeding groups* within the realm of social media, as this was also brought up in the group. There exist both *mothering* and *parenting* groups, where all parenting-related questions can be asked (eg, teething, formula feeding, and sleep training). The social media group used for this study was a *probreastfeeding* group, by self-indication. Administrators deterred other topics unless they were related to breastfeeding. There were breastfeeding experts in this group as well as other mothers who had successfully breastfed, creating somewhat of a natural community of practice. Mothers reported the probreastfeeding social media group being known for its strong and accurate advice, whereas other broader groups were designated as proformula and shamed breastfeeding mothers. Although most women had positive experiences with social media groups, not all experiences were positive, and there exists great variability in the ability of a group to support breastfeeding. This may shed light on the importance of alignment to group values as a necessary ingredient for or predictor of positive experiences and support for breastfeeding within the social media group. Furthermore, this shows the need for social media groups that are dedicated to different parenting topics such as breastfeeding or nutrition.

Although this study has discussed breastfeeding mothers’ perceptions on the role of a *probreastfeeding* social media group for breastfeeding support, it is by no means exhaustive of all social media groups or of all breastfeeding mothers. However, now that qualitative data have been explored and it is known that social media groups could positively influence breastfeeding attitudes, opportunities to further explore this topic are immense. One area not explored in this study is unintended consequences of these types of social media groups. As this was a cross-sectional design, it is not best to answer this type of question. Future research should explore unintended consequences in a holistic manner. It is clear that there is a need for future research to design and implement interventions using social media groups in breastfeeding mothers to see if associations with standard breastfeeding outcomes exist. Future research should focus on the utilization of social media groups as a way to reach breastfeeding mothers from a clinical setting (ie, hospitals, lactation consultants, and postpartum support groups). As telemedicine has arisen in recent years, one cannot help but wonder if a model of care for breastfeeding mothers using peer support in a social media group format can be designed, implemented, and tested for efficacy. It is imperative to support breastfeeding mothers throughout the duration of the breastfeeding relationship; social media groups show promise as an effective way to do so.

There are many strengths of this study, including the participation of breastfeeding mothers, who can be a hard-to-reach or sensitive population; the innovation approach using online FGDs; and the uniqueness of the study phenomenon. Deggs and Woodyatt have published the strengths and opportunities of online FGDs, along with others [38-42]. Furthermore, the utilization of this methodology for this study is available online for review, which describes recommendations for including mothers in qualitative studies to yield rich data [30]. As mentioned, this study shines light on the use of social media groups as a tool for supporting breastfeeding mothers. However, although there are numerous strengths to this approach, there are also limitations to this study that must be considered.

### Limitations

As this was an exploratory study with a small qualitative sample from a group of mothers located mainly in the southeast, the results may not be generalizable to all breastfeeding mothers who use social media. The smaller sample size was intentional, as qualitative studies are usually small in number because of their in-depth nature. However, there are some sociodemographic characteristics of this sample, which limit the generalizability of the study. These include a higher-than-average college education rate (30% have a master’s or professional degree) and a high white percentage (86%). In addition, as online FGDs and one-on-one interviews rely on the individuals’ perceptions and experiences of social media use and breastfeeding, these perceptions are dependent on sample selection. For mothers who were not currently breastfeeding, their reflection about the social media group was retrospective, the content of the social media group could have influenced them differently, which is another limitation to the study.

### Conclusions

Our study shows that social media can positively influence breastfeeding related attitudes, knowledge, and behavior. The overarching theme of community reigned in this research, with a strong emphasis on social media groups as a way to normalize breastfeeding, to empower breastfeeding mothers, to serve as a resource for women, and to share experiences related to breastfeeding. Although findings from this study are novel to the field, they reflect broader studies that identify social media as a way to reach mothers and impact their parenting-related attitudes, beliefs, and behaviors [17,20,35,37]. Furthermore, this study specifically addresses a gap in the literature on how social media can influence infant-feeding practices and, specifically, breastfeeding [20]. In general, interaction with the social media group was reported to have a positive impact on the breastfeeding journey by way of all the main themes. As this study shows social media can have a profound impact on breastfeeding mothers in a positive way, ways to catalyze a shift in the way women receive health information must be jump-started. Future research should focus on how health care professionals and organizations can use social media groups to positively influence breastfeeding attitudes, knowledge, and behaviors to increase exclusively breastfeeding duration and decrease barriers or stigmas associated with breastfeeding, leading to better quality of life for mother-infant dyads, including both physical and mental health outcomes.

## Abbreviations

FGD: focus group discussions
IBCLC: International Board Certified Lactation Consultant
MICH: maternal, infant, and child health
SNS: social networking sites
